# Investigation of cortical thickness and volume during spontaneous attacks of migraine without aura: a 3-Tesla MRI study

**DOI:** 10.1186/s10194-021-01312-9

**Published:** 2021-08-21

**Authors:** Faisal Mohammad Amin, Roberto De Icco, Mohammad Al-Mahdi Al-Karagholi, Jayachandra Raghava, Frauke Wolfram, Henrik B. W. Larsson, Messoud Ashina

**Affiliations:** 1 Danish Headache Center, Department of Neurology, Faculty of Health and Medical Sciences, Rigshospitalet Glostrup, University of Copenhagen, Valdemar Hansens Vej 5, 2600 Glostrup, Denmark; 2 Headache Science & Neurorehabilitation Center, IRCCS Mondino Foundation, Pavia, Italy; 3 Department of Brain and Behavioral Sciences, University of Pavia, Pavia, Italy; 4 Functional Imaging Unit, Department of Clinical Physiology, Nuclear Medicine and PET,Faculty of Health and Medical Sciences, Rigshospitalet, University of Copenhagen, Glostrup, Denmark; 5 Centre for Neuropsychiatric Schizophrenia Research, CNSR and Centre for Clinical Intervention and Neuropsychiatric Schizophrenia Research, CINS, Mental Health Centre Glostrup, University of Copenhagen, 2600 Glostrup, Denmark; 6 Department of Radiology, Herlev-Gentofte Hospital, University of Copenhagen, Herlev, Denmark

**Keywords:** Migraine cortex, Pain cortex, Migraine brain, Cortical volume, Migraine attack

## Abstract

**Background:**

Structural imaging has revealed changes in cortical thickness in migraine patients compared to healthy controls is reported, but presence of dynamic cortical and subcortical changes during migraine attack versus inter-ictal phase is unknown. The aim of the present study was to investigate possible changes in cortical thickness during spontaneous migraine attacks. We hypothesized that pain-related cortical area would be affected during the attack compared to an inter-ictal phase.

**Methods:**

Twenty-five patients with migraine without aura underwent three-dimensional T1-weighted imaging on a 3-Tesla MRI scanner during spontaneous and untreated migraine attacks. Subsequently, 20 patients were scanned in the inter-ictal phase, while 5 patients did not show up for the inter-ictal scan. Four patients were excluded from the analysis because of bilateral migraine pain and another one patient was excluded due to technical error in the imaging. Longitudinal image processing was done using FreeSurfer. Repeated measures ANOVA was used for statistical analysis and to control for multiple comparison the level of significance was set at *p* = 0.025.

**Results:**

In a total of 15 patients, we found reduced cortical thickness of the precentral (*p* = 0.023), pericalcarine (*p* = 0.024), and temporal pole (*p* = 0.017) cortices during the attack compared to the inter-ictal phase. Cortical volume was reduced in prefrontal (*p* = 0.018) and pericalcarine (*p* = 0.017) cortices. Hippocampus volume was increased during attack (*p* = 0.007). We found no correlations between the pain side or any other clinical parameters and the reduced cortical size.

**Conclusion:**

Spontaneous migraine attacks are accompanied by transient reduced cortical thickness and volume in pain-related areas. The findings constitute a fingerprint of acute pain in migraine patients, which can be used as a possible biomarker to predict antimigraine treatment effect in future studies.

**Trial registration:**

The study was registered at ClinicalTrials.gov (NCT02202486).

## Background

Structural neuroimaging studies in migraine had focus on unveiling underlying pathophysiological mechanisms and radiological biomarkers of the disease. Several data supported the presence of cortical and sub-cortical morphological alterations in the migraine brain, although conflicting evidences were published [1]. When compared to healthy controls, migraine patients were characterized by an increased cortical thickness of somatosensory, frontal, occipital, and temporal areas [2–4]. In contrast, other studies reported a decreased thickness of frontal as well as somatosensory areas [4–6], or even no differences with healthy controls [7]. Another way to investigate the migraine brain is represented by the comparison of different phases of the migraine cycle. Resting-state functional magnetic resonance imaging was widely used in the past years to explore this topic. During spontaneous migraine attacks, an altered thalamocortical network was described, namely an increased connectivity with orbitofrontal and parietal cortices, and a decreased connectivity with primary somatosensory cortex [8]. Moreover, the hypothalamus showed dynamic connections during the migraine cycle namely a coupling with the spinal trigeminal nuclei in the inter-ictal phase, that shifts to a stronger connection with the dorsal rostral pons in the ictal phase [9]. Nonetheless, cortical thickness and volume could exhibit rapid changes when studied in different conditions, or after exposure to specific stimuli [10]. The pathophysiology of this acute modulation is not completely elucidated, and fast-adjusting processes, such as regulation of glial cells volumes, interstitial cortical space as well as synaptic elements, probably play a major role [10]. Despite these observations, evidences about morphologic changes between different phases of the migraine cycle are lacking.

The aim of this study is to investigate the morphological changes of cortical and sub-cortical brain structures during spontaneous migraine attacks of migraine without aura compared to outside of the attack. We hypothesized that migraine attacks would be accompanied by morphological changes in pain-related areas in the brain.

## Methods

### Study design and participants

Patients were recruited from 2013 to 2014 among those attending the outpatient clinic of the Danish Headache Center (Copenhagen, Denmark) and through web a page (http://www.forsoegsperson.dk). Inclusion criteria were: age 18 to 65 years; diagnosis of episodic migraine without aura according to the ICHD criteria [11]. Exclusion criteria were: history of any other primary or secondary headache (only infrequent episodic tension-type headache less than 5 days a month was allowed); pregnant or breast-feeding women; contraindication for MRI; signs or verified diagnosis of any cardiovascular or cerebrovascular disease; active psychiatric disorder and/or drug abuse.

### Procedures

The patients underwent two MRI scans during two separate sessions: session 1 (S1) was performed during a spontaneous migraine attack, while session 2 (S2) was performed in the inter-ictal period.

During S1, patients were asked to call an investigator of the study (FMA) as soon as possible after the onset of a migraine attack. All patients were previously instructed about the International Headache Society’s migraine without aura criteria [12]. After the call, patients travelled to the hospital by taxi or public transport (fare reimbursed) between 4 and 24 h from attack onset. After hospital arrival, the patients underwent the first brain MRI scan during the spontaneous migraine attack. Patients were not allowed to treat their attacks before the MRI scan. During S2, the same patients were scanned during an inter-ictal migraine-free period. The inter-ictal period was defined as no headache in the 48 h, and no migraine attack in the 72 h before the MRI scan. Analgesic as well as migraine-specific drugs were not allowed in the 48 h before and 24 h after S1 and S2. All T1-weighted images were assessed by a neuroradiologist (FW) for pathological abnormalities, which were not found in any of the patients included in the present study.

### MRI data analysis

We obtained the MRI recordings from a Philips Achieva 3 T MRI scanner (Philips Healthcare, Best, Netherlands) using a 32-channel SENSE head coil. To minimize motion artefacts due to head movement, we placed foam pads in both temple regions in the head coil. The three-dimensional high-resolution T1-weighted MP-RAGE (magnetization-prepared rapid gradient-echo) images of the brain were acquired with the following parameters: repetition time 6.9 ms, echo time 2.78 ms, field-of-view 263 mm × 281 mm × 150 mm, matrix size 256 mm × 256 mm, 137 sagittal slices, and voxel size 1.1 mm × 1.1. mm × 1.1 mm. FreeSurfer version 5.3.0 was used to process the acquired T1-weighted images using the longitudinal processing pipeline described in detail elsewhere [11, 13, 14]. First, all scans were processed cross-sectionally at each time point with the default automated processing pipeline that includes motion correction, intensity normalization, skull stripping, coregistration, segmentation, spherical registration and cortical/subcortical parcellations [15, 16]. Then, an unbiased within-subject template is generated for each participant by processing the scans from both the time points. Several following processing steps, such as brain extraction, atlas registration, generation of cortical surface maps and parcellations are then initialized from the within-subject template to obtain longitudinally processed data. This significantly increased sensitivity, reliability, statistical power and ensures inverse consistency [14]. The Desikan–Killiany atlas [17] in FreeSurfer was used to extract regional measures of cortical thickness and volume [16, 18].

### Statistical analysis

The statistical analysis was performed with SPSS version 21.0 for Windows. All clinical and demographic variables and cortical thickness values, including volume of cortical and subcortical structures showed a normal distribution using the Kolmogorov-Smirnov test. The analysis was performed with a two factors ANOVA for repeated measures: factor SESSION (2 levels: S1 scan vs. S2 scan) and factor SIDE (pain side vs. non-pain side), followed by a post-hoc correction for multiple comparison according to Bonferroni. Association between clinical and demographic variables and MRI data were performed by mean of specifically designed linear regression models. The experimental conditions before S1 and S2 scans were compared with a Student t-test for paired samples. Continuous variables are presented as “mean +/- standard deviation”, while categorical variables are presented as “absolute numbers with percentages”. Finally, considering that every ROI was tested twice (ANOVA and correlation analysis), to further control for multiple comparison we set the level of significance at alpha = 0.025.

## Results

### Study population

Twenty-five patients were enrolled in the study and completed the S1 session (i.e. all 25 patients were scanned during on-going migraine headache). Of these, 10 patients were excluded for the following reasons: 5 patients were lost to follow-up and did not complete the S2 session; 4 patients had a bilateral migraine attack during the S1 scan; 1 patient was excluded because it was impossible to analyze the S1 MRI scan for the presence of movement artefacts.

A complete dataset was available for 15 patients (age 40.1 ± 10.3 years, 13 female, height 169.3 ± 5.9 cm, weight 86.3 ± 11.1 Kg, 12 right-handed). The study population had an average disease duration of 19.7 ± 10.1 years, and a migraine frequency of 3.3 ± 1.9 days per month. Four patients (26.7%) were on a stable preventive drug therapy during the overall study period (data are summarized in Table 1).

**Table 1:** Clinical and demographic features of study population

		Study Population
N		15
Age (years)		40.1 ± 10.3
Female sex		13 (86.7%)
Height (cm)		169.3 ± 5.9
Weight (kg)		86.3 ± 11.1
Right handedness		12 (80.0%)
Disease duration (years)		19.7 ± 10.1
Number of migraine attack per month		3.3 ± 1.9
Number of migraine days per month		6.6 ± 3.7
Migraine prophylaxis		4 (26.7%)
Usual side of migraine	Left	8 (53.3%)
	Right	3 (20.0%)
	Shifting	4 (26.7%)
Days between S1 and S2		30.2 ± 15.9

Legend: S1: MRI scan performed during spontaneous migraine attack; S2: MRI scan performed during inter-ictal period

S1 and S2 scans were performed 30.2 ± 15.9 days apart. The number of hours of sleep before MRI scan (*p* = 0.954) and the days elapsed since the last menses (*p* = 0.145) were comparable between S1 and S2 MRI sessions. In contrast, the days elapsed since last migraine attacks were higher before S1 scan (*p* = 0.015) (Table 2).

**Table 2:** Comparison of experimental conditions between S1 and S2 scans, and clinical phenotype of migraine attack recorded at S1

		S1 Spontaneous migraine attack	S2 Inter-ictal period	*p*-value
N		15	15	–
Days elapsed since last migraine attack		20.1 ± 16.3	9.3 ± 6.8	0.015
Sleep hours before MRI scan		6.8 ± 2.0	6.9 ± 1.3	0.954
Days elapsed since last menses (for female patients, *N* = 13)		29.1 ± 27.3	42.3 ± 49.1	0.145
Side of migraine attack during S1	Left	8 (53.3%)		
	Right	7 (46.7%)		
Hours from migraine onset to scan		7.9 ± 3.7		
NRS of migraine pain during S1		6.8 ± 2.1		
Throbbing pain		14 (93.3%)		
Nausea		15 (100.0%)		
Vomit		2 (13.3%)		
Aggravated by physical activity		13 (86.7%)		
Photophobia		11 (73.3%)		
Phonophobia		5 (33.3%)		

Legend: S1: MRI scan performed during spontaneous migraine attack; S2: MRI scan performed during inter-ictal period; NRS: nociceptive rating scale

S1 scan was performed after 7.9 ± 3.7 h from migraine onset. The clinical phenotype of the migraine attack recorded during S1 are summarized in Table 2.

### Structural changes during attack (S1 scan) compared to the inter-ictal period (S2 scan)

**Fig. 1:**
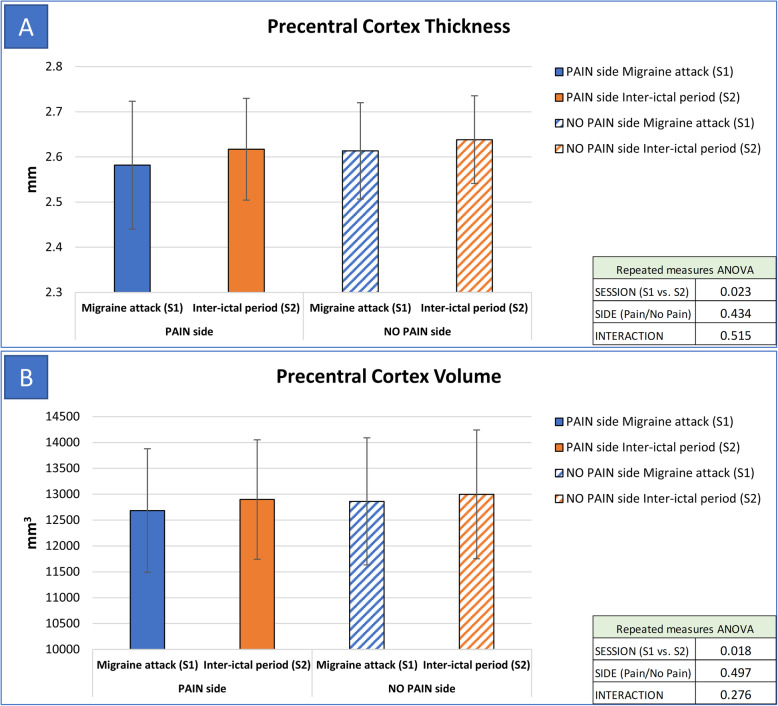
Comparison of precentral cortex thickness and volume between S1 and S2 scans. Legend: S1: MRI scan performed during spontaneous migraine attack; S2: MRI scan performed during inter-ictal period; PAIN side refers to the side of spontaneous migraine attack recorded at T1. Panel A: thickness of precentral cortex was significantly lower during S1 (factor SESSION: *p* = 0.023), without significant association with the pain side (factor SIDE: *p* = 0.434, interaction SESSIONxSIDE: *p* = 0.515). Panel B: volume of precentral cortex was significantly lower during S1 (factor SESSION: *p* = 0.018), without significant association with the pain side (factor SIDE: *p* = 0.497, interaction SESSIONxSIDE: *p* = 0.276)

**Fig. 2:**
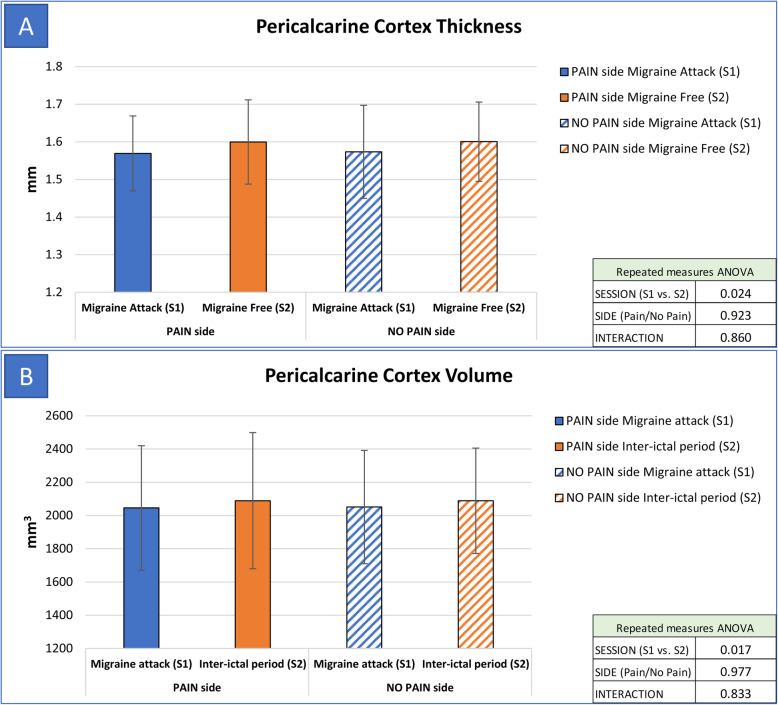
Comparison of pericalcarine cortex thickness and volume between S1 and S2 scans. Legend: S1: MRI scan performed during spontaneous migraine attack; S2: MRI scan performed during inter-ictal period; PAIN side refers to the side of spontaneous migraine attack recorded at T1. Panel A: thickness of pericalcarine cortex was significantly lower during S1 (factor SESSION: *p* = 0.024), without significant association with the pain side (factor SIDE: *p* = 0.923, interaction SESSIONxSIDE: *p* = 0.860). Panel B: volume of pericalcarine cortex was significantly lower during S1 (factor SESSION: *p* = 0.017), without significant association with the pain side (factor SIDE: *p* = 0.977, interaction SESSIONxSIDE: *p* = 0.833)

When compared to S2 (inter-ictal scan), during S1 (migraine attack scan) we found a significant reduction of thickness and volume of precentral cortex (*p* = 0.023 for thickness, and *p* = 0.018 for volume – Fig. 1) and pericalcarine cortex (*p* = 0.024 for thickness, and *p* = 0.017 for volume – Fig. 2). Also, the temporal pole cortex thickness was significantly lower at S1 when compared to S2 (*p* = 0.017). Regarding subcortical structures, we found an increase of hippocampus volume during S1 (*p* = 0.007).

### Correlation of structural changes with pain

For all the results described above, we did not find a significant association with the side of migraine attack pain. Only for the temporal pole cortex thickness there was a significant interaction session and side (*p* = 0.009), but the post-hoc analysis was not statistically significant. Descriptively, it showed a trend toward a reduction of its thickness ipsilaterally to the migraine attack pain side (*p* = 0.080), and toward an increase of thickness contralaterally to the pain side (*p* = 0.056). Exploratively, a linear regression model analysis showed a positive correlation between the number of days elapsed since the last migraine attack and the rostral anterior cingulate cortex thickness ipsilateral to the migraine pain side recorded during S1 (R^2^ = 0.727). When corrected for age, sex, disease duration, and migraine frequency, the thickness of rostral anterior cingulate cortex showed an increase of 0.014 mm for every day elapsed since last migraine attack (*p* = 0.014) (Table 3).

**Table 3:** Multivariate linear regression model; dependent Variable: rostral anterior cingulate cortex thickness ipsilateral to the migraine pain side recorded during S1

	Non standardized coefficients		Standardized coefficients	t	*p*-value	95% confidence interval fo B	
	B	Standard deviation error	Beta			Lower limit	Upper limit
Constant	2.885	0.440		6.562	0.000	1.890	3.879
Gender	−0.250	0.144	−0.328	−1.732	0.117	−0.577	0.077
Age	0.003	0.008	0.093	0.314	0.761	−0.015	0.020
Disease duration (years)	0.015	0.000	0.581	1.841	0.099	−0.004	0.034
Number of migraine attacks per month	−0.027	0.040	−0.187	−0.667	0.521	−0.117	0.064
Days elapsed since last migraine attack	0.014	0.005	0.820	3.034	0.014	0.004	0.024

R^2^ of multivariate linear regression model: 0.727

## Discussion

This is the first study specifically designed to evaluate whether cortical and sub-cortical brain structures show morphological changes during a spontaneous migraine attack when compared to the inter-ictal period. The main findings of our study are that thickness and volume of precentral and pericalcarine cortices, and thickness of the temporal pole cortex were reduced during a spontaneous migraine attack. At subcortical level, the hippocampus volume was increased during migraine attack. All these structures are involved in pain modulation and perception as part of the pain matrix [19, 20].

Cortical thickness and volume are measures of the size of brain cortex, which is progressively reduced with the age. However, cortical thickness can also be modified by various stimuli, including for instance transcranial magnetic stimulation (TMS) [21] and electroconvulsive therapy (ECT) [22]. A longitudinal MRI study of ECT in depressive patients reported increased cortical thickness immediately after ECT, but which was normalized to baseline after a 6 months follow-up period [22]. Thus, transient changes in cortical thickness can also occur. In the present study, cortical thickness was reduced during the attacks compared to inter-ictal phase in the patients. Due to the lack of a healthy control group it is not known whether the cortical thickness in the inter-ictal phase was normal. However, previous studies comparing the inter-ictal phase in migraine patients with healthy controls reported reduced cortical volume of several areas, including the prefrontal cortex [23]. Moreover, there seems to be a relationship between the total load of attacks during life and decreased gray matter volume [24–29], suggesting that reduced cortical thickness may be a consequence of attacks. The thickness of the temporal pole [30] and pericalcarine cortex [31] is suggested to play an important role in migraine. Compared to healthy controls, Coppola and colleagues reported ictal increase and interictal reduction of gray matter density of the temporal pole in migraine patients [32]. However, this study was not longitudinal, but investigated two groups of patients. We cannot explain the opposite findings in this region. Despite the discrepancy, it is interesting that the change in the temporal pole was reported among several investigated areas. Coppola and colleagues defined the ictal phase as 12 h before or after beginning of an attack, while in our study none of the patients were scanned before onset of headache attack. The timing of the scans may be plausible explanation of the discrepancy. The mean time between the attack and inter-ictal phase scans was 30 days with the shortest interval of 12 days, but there was no correlation between cortical thickness or volume and the time between the scans. It is therefore highly likely that the changes in thickness was rapid onset and short-lasting, which can reflect short-term brain morphology but also changes in blood flow appearing as cortical thickness changes [33]. In either case, a reduced activity of these areas relative to other areas of the pain network would be expected. The dorsolateral prefrontal cortex has an inhibitory role in pain pathways [34] and TMS of the prefrontal cortex is an effective therapy in chronic migraine [35]. In depressive patients TMS induced increase of cortical thickness, which was related to good effect of the therapy [21]. In addition to its efficacy in chronic migraine [36], TMS can also be used as acute treatment of the migraine attack. A randomized double-blind, parallel-group, sham-controlled trial of 164 patients reported significant higher responder rates after 2 h in the group treated with single-pulse TMS in the occipital region (visual cortex) compared with sham in the same region [37]. Like the prefrontal cortex, the visual cortex may have an inhibitory role in pain processing, which is affected during migraine attacks. It is known that cortical thickness of the visual cortex is reduced inter-ictal [3, 4, 38] and here we report a further reduction during the attack.

The role of hippocampus and its morphological alterations were also evaluated in migraine patients [39]. It is noteworthy that hippocampal volume seemed to strictly relate to headache frequency, as it was greater in low-frequency migraine when compared to high-frequency migraine [40]. Moreover, hippocampal volume correlated with the migraine frequency, showing a positive plastic adaption at low migraine frequency, and negative adaptation at higher headache frequency [41].

The biological significance of the structural changes observed in the present study is yet to be elucidated but could be a consequence of hypoperfusion rather than shrinking of the neurons and glial cells. In this view, it is worth noting that even if these brain areas showed a significant modulation during a spontaneous migraine attack in our cohort of patients, we were not able to find an association with the migraine pain side as well as significant correlations with other clinical features of the disease. For these reasons, our results must be interpreted with caution, because they could represent a fast plastic adaptation to a non-specific stimulus, more than a specific reaction of the brain to the migraine attack. Reduced cortical thickness of the visual cortex and the prefrontal cortex may most likely reflect trigeminovascular and general pain respectively [42, 43]. In which order of the migraine cycle, cortical thickness change occurs is yet unknown, as we have no pre-ictal MRI scans in our patients. Repeated MRI scans in the individual patients may explain this in future.

Another interesting, but explorative finding was that the cortical thickness recorded during the spontaneous migraine attack of rostral anterior cingulate cortex ipsilateral to the migraine pain side, positively correlated with number of days elapsed since the previous migraine attack. This result was consistent after correction for age, sex, disease duration, and migraine frequency. The plastic adaptation of the cingulate cortex is not specific to migraine as it was reported in several pain and non-pain conditions [6]. In our cohort, the gradual increase in thickness of the cingulate cortex could represent a compensatory mechanism that starts and develops after a painful stimulus. On the other hand, the anterior cingulate cortex thickness was not significantly reduced during a spontaneous migraine attack, and for this reason this result must be interpret with caution.

The low sample size of the present cohort limits further analyses in subgroups of patients (e.g. those with nausea versus without nausea etc.). Moreover, this study was designed to investigate the pain phase, why we waited at least 4 h from onset to scan. Thus, we cannot examined the early phase of the attack which is a limitation of this study.

## Conclusions

We have presented the first study on morphological MRI changes during spontaneous migraine attacks compared to the inter-ictal phase. Acute attacks are accompanied by reduced cortical thickness and volume in pain and previously migraine-related areas. Although, we do not ascribe present findings as being migraine specific, these findings constitute a fingerprint of acute pain in migraine patients. In future studies, this fingerprint can be used as a biomarker in order to evaluate antimigraine treatment effect.

## Data Availability

The data that support the findings of this study are available, but restrictions apply to the availability of these data, which were used under license for the current study, and so are not publicly available. Data are however available from the authors upon reasonable request and with permission of data protection department of Rigshospitalet, Copenhagen, Denmark.

## Abbreviations

ANOVA: Analysis of variance
ECT: Electroconvulsive therapy
ICHD: International Classification of Headache Disorders
MP-RAGE: Magnetization-prepared rapid gradient-echo
MRI: Magnetic resonance imaging
TMS: Transcranial magnetic stimulation
