# Correction: Investigation of cortical thickness and volume during spontaneous attacks of migraine without aura: a 3-Tesla MRI study

**DOI:** 10.1186/s10194-023-01549-6

**Published:** 2023-03-06

**Authors:** Faisal Mohammad Amin, Roberto De Icco, Mohammad Al-Mahdi Al-Karagholi, Jayachandra M. Raghava, Frauke Wolfram, Henrik B. W. Larsson, Messoud Ashina

**Affiliations:** 1 Danish Headache Center, Department of Neurology, Faculty of Health and Medical Sciences, Rigshospitalet Glostrup, University of Copenhagen, Valdemar Hansens Vej 5, 2600 Glostrup, Denmark; 2 Headache Science & Neurorehabilitation Center, IRCCS Mondino Foundation, Pavia, Italy; 3 Department of Brain and Behavioral Sciences, University of Pavia, Pavia, Italy; 4 Functional Imaging Unit, Department of Clinical Physiology, Nuclear Medicine and PET, Faculty of Health and Medical Sciences, Rigshospitalet, University of Copenhagen, Glostrup, Denmark; 5 Centre for Neuropsychiatric Schizophrenia Research, CNSR and Centre for Clinical Intervention and Neuropsychiatric Schizophrenia Research, CINS, Mental Health Centre Glostrup, University of Copenhagen, 2600 Glostrup, Denmark; 6 Department of Radiology, Herlev-Gentofte Hospital, University of Copenhagen, Herlev, Denmark

**Correction: J Headache Pain 22, 98 (2021)**

**https://doi.org/10.1186/s10194-021-01312-9**

Following publication of the original article [1], the author group has identified an error in the author name of Jayachandra M. Raghava.

The incorrect author name is: Jayachandra Raghava

The correct author name is: Jayachandra M. Raghava

The author group has been updated above and the original article [1] has been corrected.
